# Supplementary material for: Dynamic and intricate regulation by the Csr sRNAs in the Arctic Pseudoalteromonas fuliginea
Source: Commun Biol. 2025 Mar 5;8:369. doi: 10.1038/s42003-025-07780-y (PMC11882849; doi:10.1038/s42003-025-07780-y)
Supplement: Supplementary file 1 — Supplementary Information [file 42003_2025_7780_MOESM1_ESM.pdf]

Supplementary Figures

Dynamic and intricate regulation by the Csr sRNAs in the Arctic *Pseudoalteromonas fuliginea*

Zedong Duan[1,2], Li Liao[1,2]*, Tingyi Lai[1,2], Ruyi Yang[2,3], Jin Zhang[2], Bo Chen[2]

[1]Key Laboratory of Polar Ecosystem and Climate Change, Ministry of Education; Shanghai Key Laboratory of Polar Life and Environment Sciences; and School of Oceanography, Shanghai Jiao Tong University, Shanghai, 200030, China.
[2]Key Laboratory for Polar Science, Ministry of Natural Resources, Polar Research Institute of China, Shanghai, China
[3]School of Health Science and Engineering, University of Shanghai for Science and Technology, Shanghai, 200093, China.

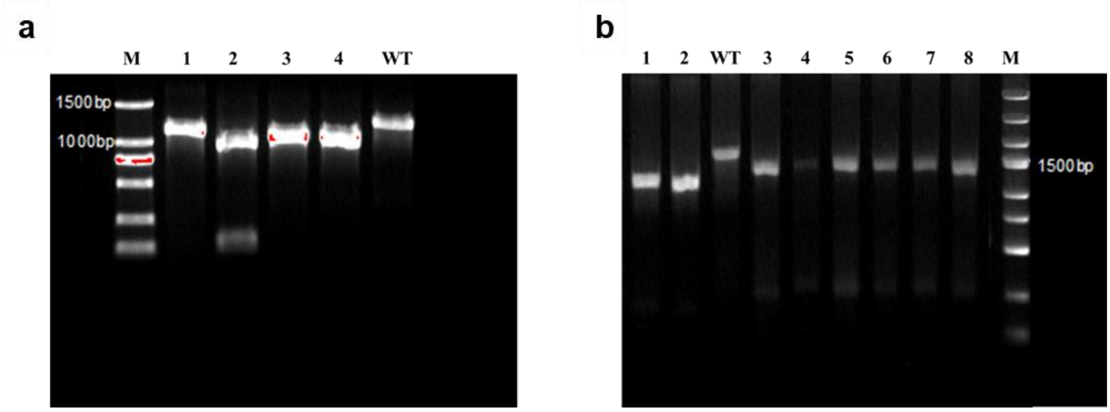

Supplementary Fig. 1. Confirmation of deletion of Pf2 and Pf3 in *P. fuliginea* BSW20308. (**a**) PCR detection of the Pf2 knockout plasmids conjugation transformation and Pf2 knockout mutants. M, DNA Marker. WT, BSW20308 wild-type strain. Two sets of primers that are specific to the pK18-mobsacB-Ery plasmids (Ery-F/ Ery-R and SacB-F/ SacB-R) were used to verify the successful entry of the Pf2 knockout plasmid into the *P. fuliginea* BSW20308 (lanes 1 and 2). Lanes 3 and 4, the independent colonies that were verified the knockout of Pf2. (**b**) PCR detection of the Pf3 knockout plasmids conjugation transformation (lanes 1 and 2) and Pf3 knockout mutants (lanes 3 to 8).

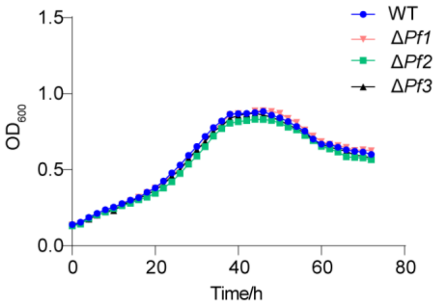

Supplementary Fig. 2. Growth curves of Pf sRNA mutants and the wild type. Growth curves of the wild type (WT) and single sRNA mutant strains (Δ*Pf1*, Δ*Pf2*, Δ*Pf3*) were monitored over time.

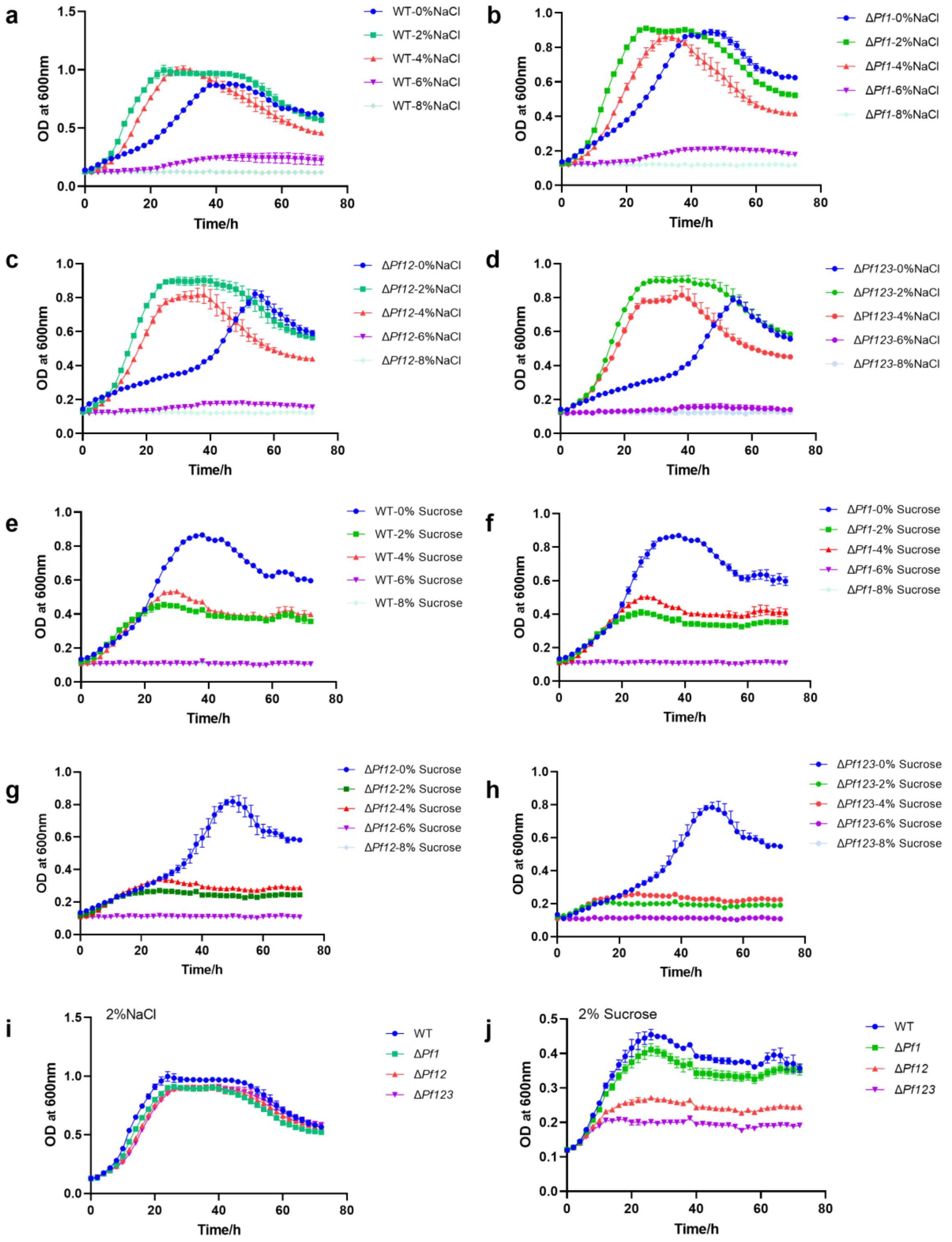

Supplementary Fig. 3. Growth curves of wild-type and Pf sRNA mutants under various salinities and sucrose concentrations. (**a-d**) Growth curves of wild-type and Pf sRNA mutants under various salinities concentrations. (**e-h**) Growth curves of wild-type and Pf sRNA mutants under various sucrose concentrations. (**i-j**) Growth curves of wild-type and Pf sRNA mutants under 2% NaCl and 2% sucrose.

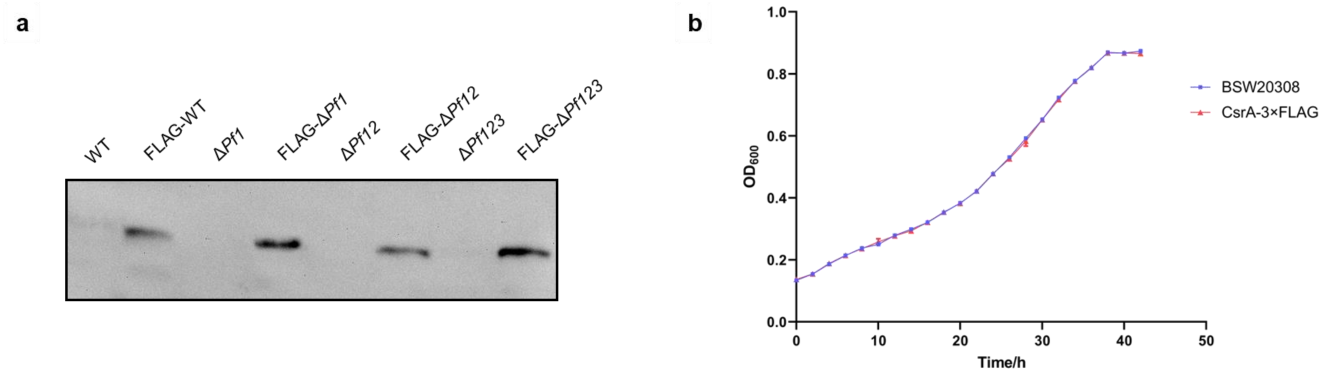

Supplementary Fig. 4. The expression of *P. fuliginea* CsrA and growth curves in WT, Δ*Pf1*, Δ*Pf12* and Δ*Pf123*. (**a**) Western blot analysis of CsrA-3xFLAG expression in WT, Δ*Pf1*, Δ*Pf12* and Δ*Pf123*. Untagged strains were treated as control. (**b**) Growth curves for *P. fuliginea* BSW20308 and CsrA-3xFLAG tagged strains grow in 2216E medium in duplicate. Error bars are mean ± s.e.m.

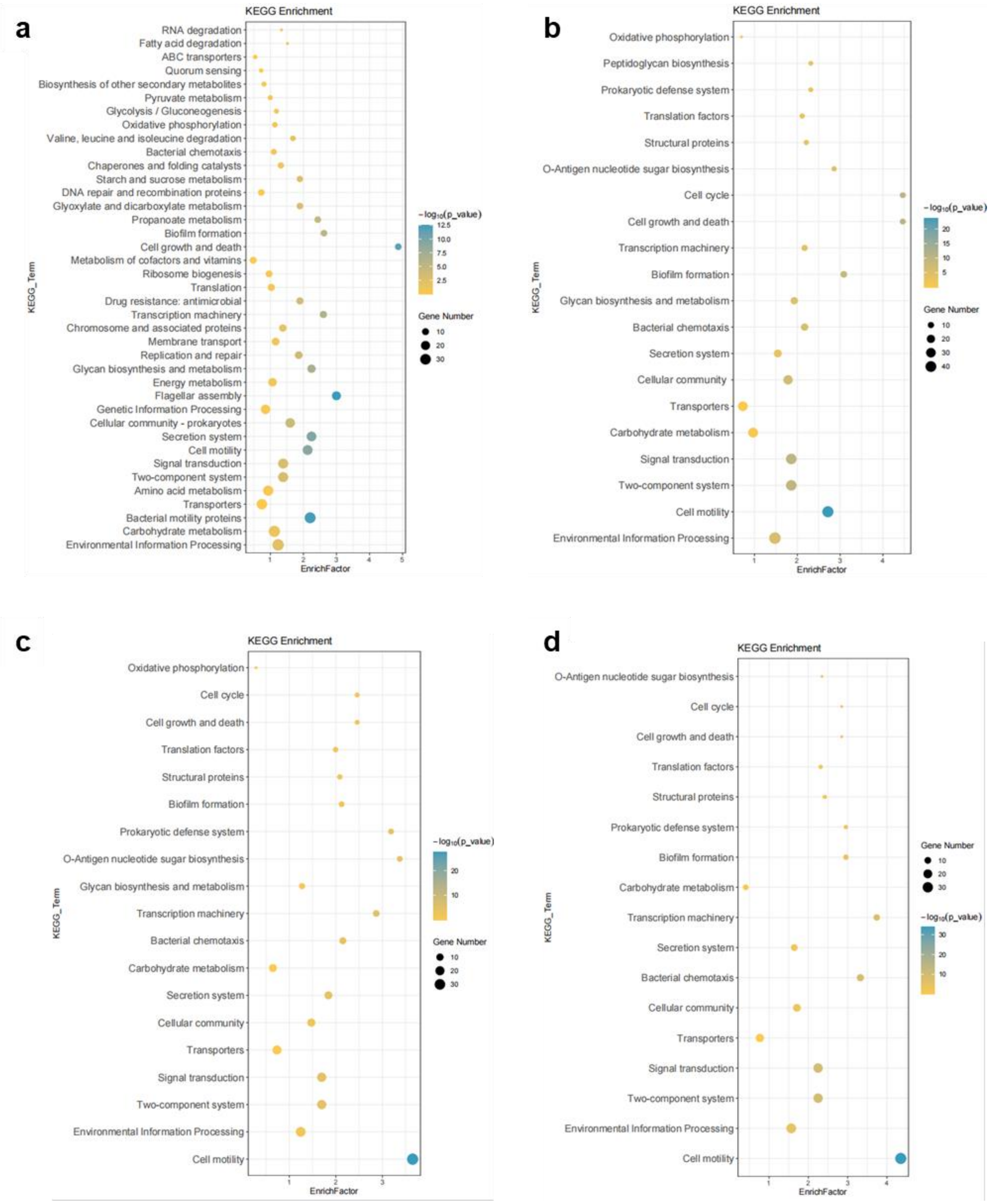

Supplementary Fig. 5. Functional annotation of enriched genes that were associated with CsrA RIP-seq peaks in WT (a), Δ*Pf1* (b), Δ*Pf12* (c), and Δ*Pf123* (d) mutants

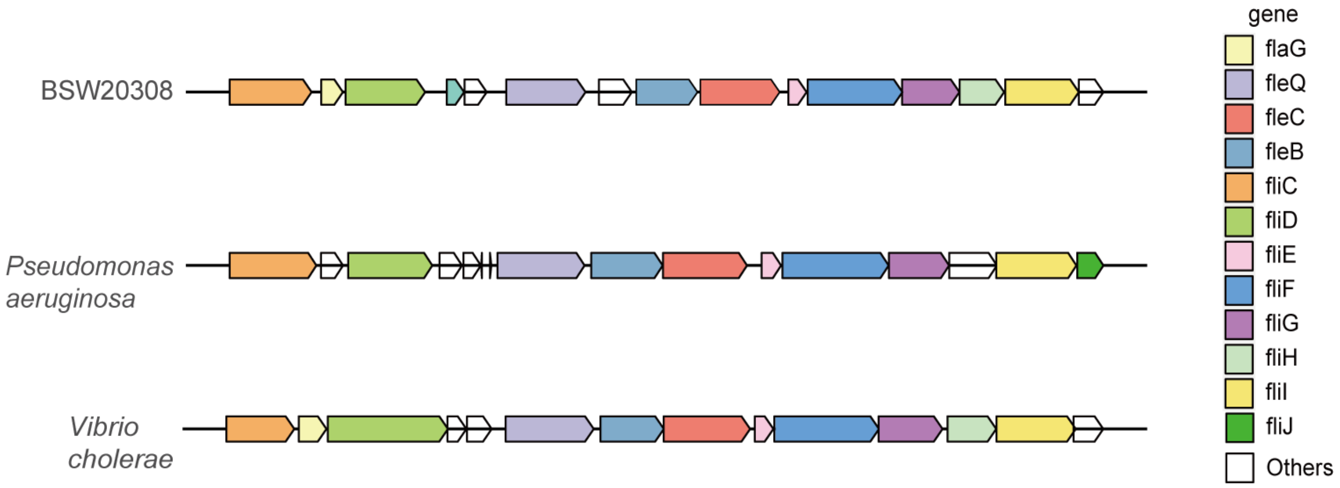

Supplementary Fig. 6. **Flagellar synthesis gene cluster of *P. fuliginea* compared with those from *Pseudomonas aeruginosa* and *Vibrio Cholerae***. The same color represents homologous genes, and genes with unknown functions are coloured white.

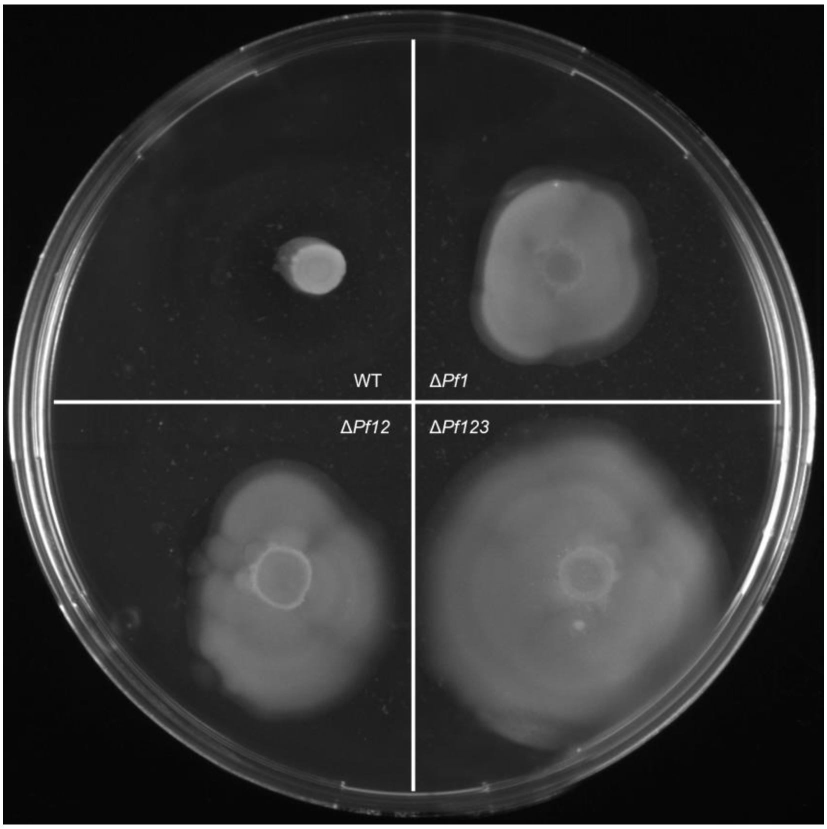

Supplementary Fig. 7. Motility assay of wild-type (WT) and mutant strains (Δ*Pf1*, Δ*Pf12*, and Δ*Pf123*) on a single soft-agar plate. The motility of each strain was evaluated on a single 2216E plate containing 0.3% agar to minimize inter-plate variability.

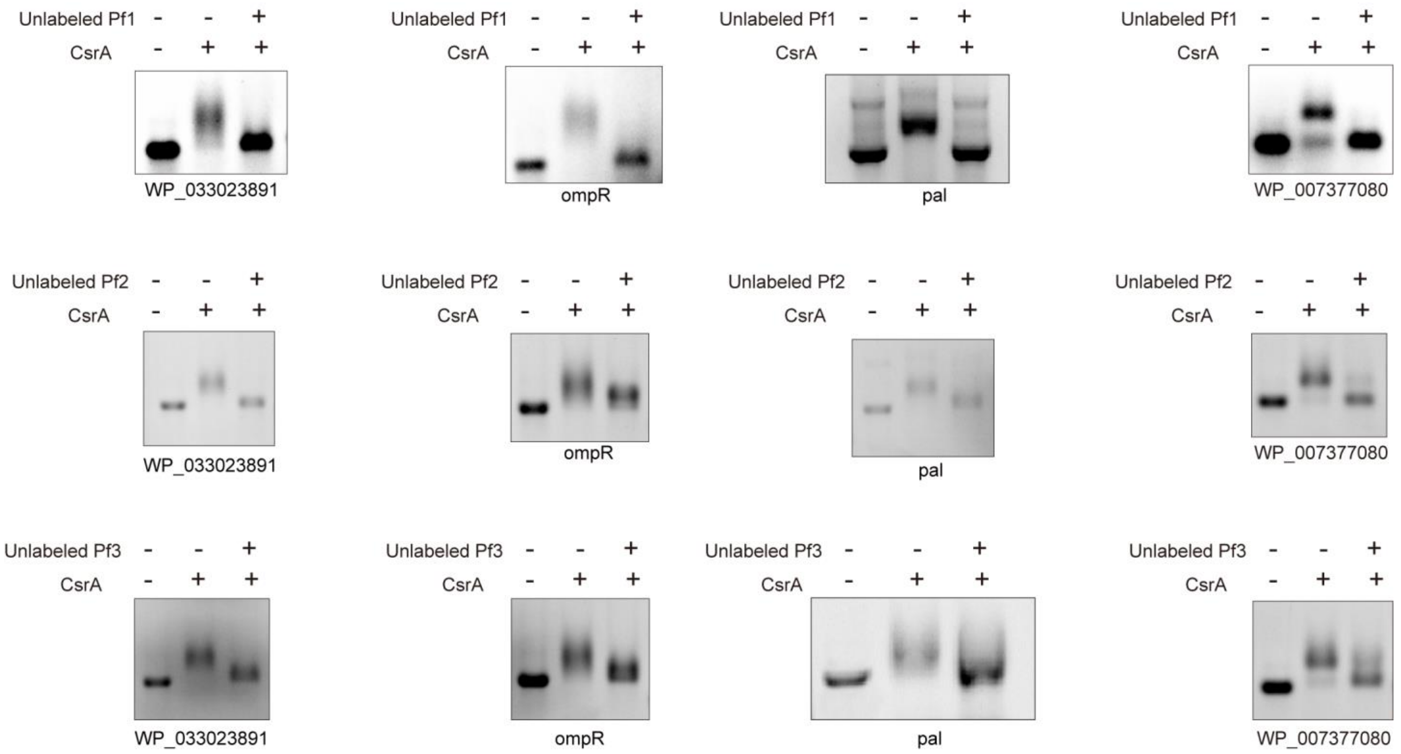

Supplementary Fig. 8. **Pf sRNAs interfered with the interaction between CsrA and its target mRNAs**. Electrophoretic mobility shift assays (EMSAs) were performed to assess the effects of unlabeled *Pf* sRNAs (*Pf1*, *Pf2*, and *Pf3*) on the binding of CsrA to its target mRNAs (*WP_033023891*, *ompR*, *pal*, and *WP_007377080*). The presence (+) or absence (−) of unlabeled *Pf* sRNAs and CsrA is indicated above each panel.

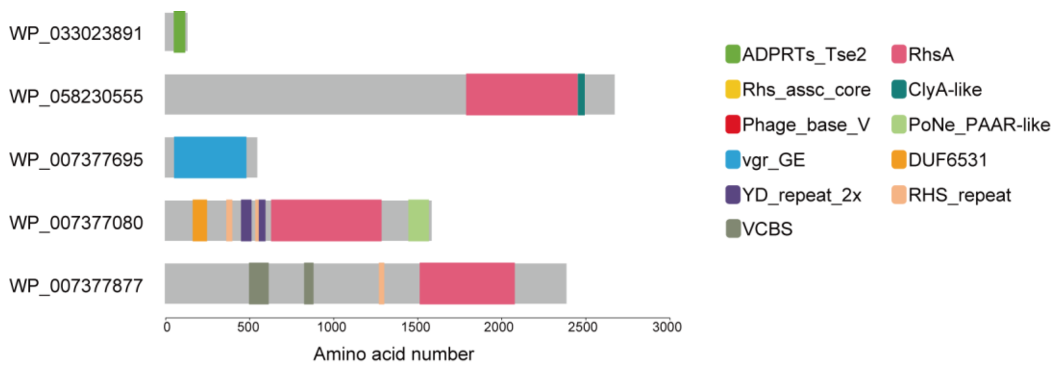

Supplementary Fig. 9. Conserved domains of the putative T6SS effectors. The diagram depicts the conserved domain architecture of five putative T6SS effector proteins (WP_033023891, WP_058230555, WP_007377695, WP_007377080, and WP_007377877). The length of each bar corresponds to the number of amino acids, and the color-coded regions represent specific conserved domains identified in the sequences.

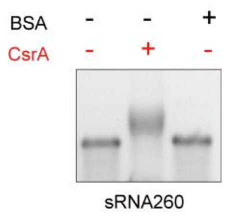

Supplementary Fig. 10. **The interaction between sRNA260 and CsrA protein.** EMSA of 1 μM CsrA binding to sRNA260 with 1 μM BSA as the control.

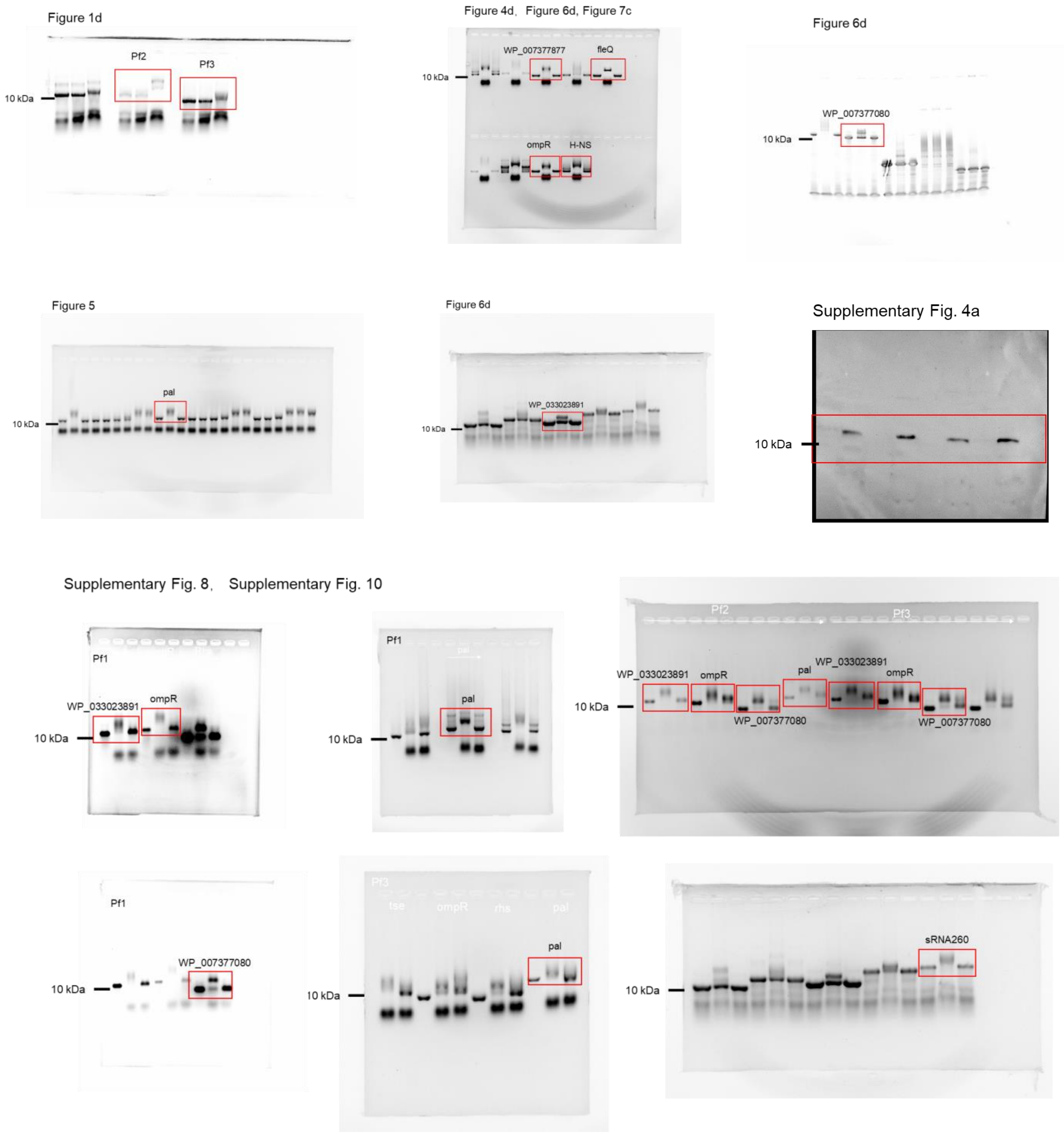

Supplementary Fig. 11. The whole blot and gel images in the study.
