# Supplementary material for: Dynamic and intricate regulation by the Csr sRNAs in the Arctic Pseudoalteromonas fuliginea
Source: Commun Biol. 2025 Mar 5;8:369. doi: 10.1038/s42003-025-07780-y (PMC11882849; doi:10.1038/s42003-025-07780-y)
Supplement: Supplementary file 2 — Description of Additional Supplementary File [file 42003_2025_7780_MOESM2_ESM.pdf]

Description Of Additional Supplementary File

File name: Supplementary Data 1
Description: Sequences matched with Pf1, Pf2, and Pf3 in NCBI database

File name: Supplementary Data 2
Description: Peaks significantly enriched in WT, ΔPf1, ΔPf12 and ΔPf123 strains ($q < 0.01$)

File name: Supplementary Data 3
Description: The mRNA targets detected by RIPSeq and their KEGG enrichment analysis

File name: Supplementary Data 4
Description: Detailed gene information on the cell motility,cell envelope,T6SS and biofilm formation of the CsrA targetomes

File name: Supplementary Data 5
Description: Potential specific targets of Csr system in P. fuliginea BSW20308

File name: Supplementary Data 6
Description: The sRNA targets detected by RIPSeq and their putative antisense targets

File name: Supplementary Data 7
Description: Bacterial strains and plasmids used in the study

File name: Supplementary Data 8
Description: Source data underlying the main figures
