# Supplementary material for: Dynamic and intricate regulation by the Csr sRNAs in the Arctic Pseudoalteromonas fuliginea
Source: Commun Biol. 2025 Mar 5;8:369. doi: 10.1038/s42003-025-07780-y (PMC11882849; doi:10.1038/s42003-025-07780-y)
Supplement: Supplementary file 11 — Reporting Summary [file 42003_2025_7780_MOESM11_ESM.pdf]

Corresponding author(s): Li Liao

Last updated by author(s): Feb 8, 2025

Reporting Summary

Nature Portfolio wishes to improve the reproducibility of the work that we publish. This form provides structure for consistency and transparency in reporting. For further information on Nature Portfolio policies, see our Editorial Policies and the Editorial Policy Checklist.

Statistics

For all statistical analyses, confirm that the following items are present in the figure legend, table legend, main text, or Methods section.

n/a	Confirmed	
☐	☒	The exact sample size (*n*) for each experimental group/condition, given as a discrete number and unit of measurement
☐	☒	A statement on whether measurements were taken from distinct samples or whether the same sample was measured repeatedly
☐	☒	The statistical test(s) used AND whether they are one- or two-sided<br>*Only common tests should be described solely by name; describe more complex techniques in the Methods section.*
☒	☐	A description of all covariates tested
☒	☐	A description of any assumptions or corrections, such as tests of normality and adjustment for multiple comparisons
☐	☒	A full description of the statistical parameters including central tendency (e.g. means) or other basic estimates (e.g. regression coefficient) AND variation (e.g. standard deviation) or associated estimates of uncertainty (e.g. confidence intervals)
☐	☒	For null hypothesis testing, the test statistic (e.g. *F*, *t*, *r*) with confidence intervals, effect sizes, degrees of freedom and *P* value noted<br>*Give P values as exact values whenever suitable.*
☒	☐	For Bayesian analysis, information on the choice of priors and Markov chain Monte Carlo settings
☒	☐	For hierarchical and complex designs, identification of the appropriate level for tests and full reporting of outcomes
☒	☐	Estimates of effect sizes (e.g. Cohen's *d*, Pearson's *r*), indicating how they were calculated

Our web collection on statistics for biologists contains articles on many of the points above.

Software and code

Policy information about availability of computer code

Data collection	Does not apply. All softwares are publically available and described in the manuscript.
Data analysis	Does not apply. All softwares are publically available and described in the manuscript.

For manuscripts utilizing custom algorithms or software that are central to the research but not yet described in published literature, software must be made available to editors and reviewers. We strongly encourage code deposition in a community repository (e.g. GitHub). See the Nature Portfolio guidelines for submitting code & software for further information.

Data

Policy information about availability of data

All manuscripts must include a data availability statement. This statement should provide the following information, where applicable:

- Accession codes, unique identifiers, or web links for publicly available datasets
- A description of any restrictions on data availability
- For clinical datasets or third party data, please ensure that the statement adheres to our policy

The raw RIP-seq data reported in this study has been uploaded in NCBI's Sequence Read Archive (SRA, accession numbers: SRR29791929 to SRR29791952) under the BioProject accession number: PRJNA1134920.

Research involving human participants, their data, or biological material

Policy information about studies with human participants or human data. See also policy information about sex, gender (identity/presentation), and sexual orientation and race, ethnicity and racism.

Reporting on sex and gender	Does not apply.
Reporting on race, ethnicity, or other socially relevant groupings	Does not apply.
Population characteristics	Does not apply.
Recruitment	Does not apply.
Ethics oversight	Does not apply.

Note that full information on the approval of the study protocol must also be provided in the manuscript.

Field-specific reporting

Please select the one below that is the best fit for your research. If you are not sure, read the appropriate sections before making your selection.

☐ Life sciences ☐ Behavioural & social sciences ☒ Ecological, evolutionary & environmental sciences

For a reference copy of the document with all sections, see nature.com/documents/nr-reporting-summary-flat.pdf

Ecological, evolutionary & environmental sciences study design

All studies must disclose on these points even when the disclosure is negative.

Study description	We investigated the targets of the CsrA protein using RIP-Seq . Furthermore, we analyzed the dynamics of the CsrA targetome by examining the differences in the CsrA protein targets among these various samples. RIP-Seq involves four strains (WT,ΔPf1, ΔPf12 and ΔPf123) with three biological replicates set for each strain.
Research sample	Pseudoalteromonas fuliginea BSW20308 is a cold-adapted bacterium isolated from the Chukchi Sea in the Arctic. We constructed ΔPf1, ΔPf12, ΔPf123 mutants based on P. fuliginea BSW20308.
Sampling strategy	Does not apply.
Data collection	The collected data are checked and recorded by authors. Data were collected from experimental results.
Timing and spatial scale	It was not necessary for this study.
Data exclusions	Quality control was conducted on the raw sequence data as described in the manuscript.
Reproducibility	In growth measurement, we established nine biological replicates for each strain. For the RIP-Seq experiment, we selected three biological replicates per strain to ensure the reliability of the experimental results. The experiments conducted in this study were repeated more than twice.
Randomization	Randomization was not necessary for this study.
Blinding	Blinding was not necessary for this study.

Did the study involve field work? ☐ Yes ☒ No

Reporting for specific materials, systems and methods

We require information from authors about some types of materials, experimental systems and methods used in many studies. Here, indicate whether each material, system or method listed is relevant to your study. If you are not sure if a list item applies to your research, read the appropriate section before selecting a response.

Materials & experimental systems

n/a	Involved in the study
☒	☐ Antibodies
☒	☐ Eukaryotic cell lines
☒	☐ Palaeontology and archaeology
☒	☐ Animals and other organisms
☒	☐ Clinical data
☒	☐ Dual use research of concern
☒	☐ Plants

Methods

n/a	Involved in the study
☒	☐ ChIP-seq
☒	☐ Flow cytometry
☒	☐ MRI-based neuroimaging

Plants

Seed stocks	Does not apply.
Novel plant genotypes	Does not apply.
Authentication	Does not apply.
